# International Medical Congress

**Published:** 1889-09

**Authors:** 


INTERNATIONAL MEDICAL CONGRESS.—The 10th International Medical Congress will be held in Berlin, commencing Aug. 4th, 1890.

